# *Letter to the Editor:* Prior Infection with Coccidioidomycosis in Nonhuman Primates and Impact on Simian Immunodeficiency Virus Disease and Vaccine Immunogenicity

**DOI:** 10.1089/aid.2021.0236

**Published:** 2022-05-11

**Authors:** Deborah H. Fuller, Megan A. O'Connor

**Affiliations:** ^1^ Department of Microbiology, University of Washington, Seattle, Washington, USA.; ^2^ Washington National Primate Research Center, Seattle, Washington, USA.

**Keywords:** animal models, HIV/SIV disease pathogenesis, nonhuman primate models, vaccines, coccidioidomycosis, pigtail macaque, simian immunodeficiency virus, vaccine immunogenicity, HIV/AIDS, Valley Fever

This letter expands on our previously published case report.^1^ In this study, we determine if prior infection with coccidioidomycosis (Valley Fever) impacted study endpoints to investigate vaccine immunogenicity during simian immunodeficiency virus (SIV) infection as a model for immunizing people living with HIV/AIDS.

Fifteen pigtail macaques were enrolled in a hepatitis B virus (HBV) vaccine study and infected with SIV as a model to determine if HIV infection may impact immunogenicity of this vaccine (Supplementary Table S1). Among these, *N* = 5 were positive for Valley Fever, received antifungal treatment, and were seronegative at the time of enrollment.

As shown in Table 1, SIV disease as measured by plasma SIV viremia, peripheral blood CD4 counts, and percentage of CD4 T cells in jejunal biopsies were similar in SIV-infected animals that were previously positive (*N* = 2) or negative (*N* = 8) for Valley Fever. In addition, the magnitude of anti-SIV T cell and antibody responses was consistent across all SIV-infected animals (Table 1). Collectively, these data indicate that prior coccidioidomycosis infection may not impact certain aspects of SIV pathogenesis nor immune responses to SIV infection.

**Table 1.** Comparison of Simian Immunodeficiency Virus Disease and Anti-Simian Immunodeficiency Virus Immunity by Valley Fever Status

	SIV+ VF−	SIV+ VF+	
Plasma SIV RNA copies/mL (Log_10_)	*n* = 8^a^	Z14333	Z15032
Peak SIV	7.72 ± 0.497	6.22	7.76
22 Weeks on ART	1.52 ± 0.106	1.48	1.68
2 Weeks post-ATI	4.09 ± 1.37	3.45	5.09
No. of CD3^+^CD4^+^ cells/mL of blood			
Pre-SIV	1562 ± 573	978	2164
Peak SIV	1009 ± 280	412	816
24 Weeks on ART	977 ± 374	450	1342
2 Weeks post-ATI	854 ± 244	N/A	1303
CD3^+^CD4^+^ cells/mL of blood (% relative to pre-SIV)			
Peak SIV	69.8 ± 25.5	42.1	29.5
24 Weeks on ART	64.3 ± 15.1	46.0	48.5
2 Weeks post-ATI	57.4 ± 11.8	N/A	47.2
%CD3^+^CD4^+^ in jejunum			
Pre-SIV	36.2 ± 17.3	44.5	68.4
25 Weeks on ART	18.9 ± 10.7	29.7	26.0
SIV-Gag SFC/10^6^ PBMC			
Peak SIV	40.6 ± 52.8	0	25.0
20 Weeks on ART	258.1 ± 397.6	510	50.0
2 Weeks post-ATI	11.2 ± 20.1	40.0	0
SIV-Env SFC/10^6^ PBMC			
Peak SIV	99.4 ± 147.9	0	30.0
20 Weeks on ART	85.0 ± 94.0	60.0	5.0
2 Weeks post-ATI	4.0 ± 7.6	0	0
SIV Env-specific IgG (μg)/mL of plasma (Log_10_)			
Peak SIV	5.29 ± 0.28	5.55	4.84
20 Weeks on ART	7.17 ± 0.22	7.41	7.46
2 Weeks post-ATI	7.15 ± 0.20	7.33	7.33

^a^ Mean ± standard deviations.

ART, antiretroviral therapy; ATI, analytic treatment interruption; N/A, not available; PBMC, peripheral blood mononuclear cells; SFC, spot forming cells; SIV, simian immunodeficiency virus; VF, Valley Fever.

To evaluate HBV vaccine immunogenicity, animals were stratified into three groups based on gender, age, and health histories, including coccidioidomycosis infection (Supplementary Table S1): (1) SIV-infected/Engerix-B vaccine, (2) SIV-infected/DNA+protein vaccine, (3) naive/DNA+protein vaccine. HBV-specific antibody responses were similar in each group and there were no notable differences in animals previously infected with coccidioidomycosis, including case study animal Z14333 (Table 2).^1^ Collectively, these data also show that pigtail macaques with prior coccidioidomycosis generate immune responses to the HBV vaccines.

**Table 2.** Comparison of Hepatitis B Virus Vaccine Immunogenicity by Valley Fever Status

	SIV+ VF−	SIV+ VF+	SIV− VF−	SIV− VF+
Engerix vaccine: HBs Ag-specific IgG (μg/mL plasma)	*n* = 4^a^	*n* = 1 Z15032	*n* = 0	*n* = 0
Post-first vaccination	7.48 ± 5.97	1.07	—	—
Post-second vaccination	109.5 ± 79.1	32.9	—	—
Post-third vaccination	141.0 ± 69.4	46.6	—	—
Experimental DNA+protein Vaccine: HBc Ag-specific IgG (μg/mL plasma)^b^	*n* = 4^a^	*n* = 1 Z14333	*n* = 2^a^	*n* = 3^a^
Post-first vaccination	1.64 ± 0.61	1.22	1.53 ± 0.22	1.06 ± 0.53
Post-second vaccination	23.2 ± 9.67	12.9	13.9 ± 7.8	19.0 ± 19.6
Post-third vaccination	215.7 ± 93.4	230.8	40.8 ± 1.68	81.7 ± 42.3

^a^ Mean ± standard deviations.

^b^ DNA and protein vaccine regimen comprised of HBV core and surface antigens and anti-CD180.

Ag, antigen; HBc, hepatitis B core; HBs, hepatitis B surface; HBV, hepatitis B virus; SIV, simian immunodeficiency virus; VF, Valley Fever.

Coccidioidomycosis relapse can occur in people living with HIV and non-HIV-infected individuals,^2,3^ but the factors associated with relapse are not fully understood. Z14333, the case study animal, was treated and monitored in accordance with recommendations for humans previously treated for coccidioidomycosis^4^ and is, to date, the only reported case of coccidioidomycosis recrudescence in an SIV-infected host, indicating that a relapse occurrence is possible but rare.

Nonhuman primates, similar to humans, are diverse in their genetics and prior histories, including exposures to the same pathogens such as cytomegalovirus (CMV), Chagas disease (*Trypanosoma cruzi*), histoplasmosis (*Histoplasma* sp.), and coccidioidomycosis (*Coccidioides*). Their susceptibility to the same diseases and close similarities to humans makes them an excellent model to study human diseases and to evaluate new vaccines and therapies. Although historical infection is always a concern and should be carefully assessed when considering animals for enrollment in infectious disease studies.

Our earlier and follow-up results reported here show that prior coccidioidomycosis infection in nonhuman primates, when properly controlled for, may not be a significant variable in HIV/AIDS pathogenesis or vaccine immunogenicity studies. However, further investigation of the impact of coccidioidomycosis infection on secondary infections, including SIV/HIV, are needed. The pigtail macaque is a valuable model for investigating HIV/AIDS and natural Valley Fever infection and supporting the development of new vaccines, diagnostics, and therapeutics for these diseases.

## Supplementary Material

Supplemental data

## References

[1] Guerriero KA, Murnane RD, Lewis TB, *et al.*: Recrudescence of natural coccidioidomycosis during combination antiretroviral therapy in a pigtail macaque experimentally infected with simian immunodeficiency virus. AIDS Res Hum Retroviruses 2021;37:505–509. 33356854 10.1089/aid.2020.0228 PMC8260887

[2] Mathew G, Smedema M, Wheat LJ, Goldman M: Relapse of coccidioidomycosis despite immune reconstitution after fluconazole secondary prophylaxis in a patient with AIDS. Mycoses 2003;46:42–44. 10.1046/j.1439-0507.2003.00833.x 12588482

[3] Catanzaro A, Galgiani JN, Levine BE, *et al.*: Fluconazole in the treatment of chronic pulmonary and nonmeningeal disseminated coccidioidomycosis. NIAID Mycoses Study Group. Am J Med 1995;98:249–256. 10.1016/s0002-9343(99)80371-4 7872341

[4] Panel on Guidelines for the Prevention and Treatment of Opportunistic Infections in Adults and Adolescents with HIV: Guidelines for the Prevention and Treatment of Opportunistic Infections in HIV-infected Adults and Adolescents: Recommendations from the Centers for Disease Control and Prevention, the National Institutes of Health, and the HIV Medicine Association of the Infectious Diseases Society of America. Available at https://clinicalinfo.hiv.gov/sites/default/files/guidelines/documents/Adult_OI.pdf, accessed December 15, 2021.

