# Supplementary material for: Letter to the Editor: Prior Infection with Coccidioidomycosis in Nonhuman Primates and Impact on Simian Immunodeficiency Virus Disease and Vaccine Immunogenicity
Source: AIDS Res Hum Retroviruses. 2022 May 11;38(5):347–9. doi: 10.1089/aid.2021.0236 (PMC9131037; doi:10.1089/aid.2021.0236)
Supplement: Supplemental data [file Supp_TableS1.docx]

| **Supplementary Table S1. Animal Characteristics** | | | | | | |
| --- | --- | --- | --- | --- | --- | --- |
| **Animal ID** | **Sex** | **Age (Yrs)** | **Weight (Kg)** | **SIV+** | **History of VF^a^** | **HBV Vaccine^B^** |
| Z16041 | M | 3.6 | 5.3 | Yes | No | DNA +protein |
| Z15329 | M | 4.0 | 5.7 | Yes | No | DNA +protein |
| Z14302 | M | 5.2 | 7.7 | Yes | N.D. | DNA +protein |
| Z15268 | M | 4.2 | 7.8 | Yes | N.D. | DNA +protein |
| Z14333 | M | 5.0 | 10.7 | Yes | Yes | DNA +protein |
| Z14362 | M | 4.9 | 9.5 | Yes | No | Engerix |
| Z15291 | M | 4.1 | 8.7 | Yes | N.D. | Engerix |
| Z15374 | M | 3.9 | 7.7 | Yes | N.D. | Engerix |
| Z15032 | M | 4.7 | 7.1 | Yes | Yes | Engerix |
| Z15182 | M | 4.4 | 10.7 | Yes | N.D. | Engerix |
| Z16277 | M | 3.2 | 5.0 | No | No | DNA +protein |
| Z14176 | M | 5.5 | 9.4 | No | Yes | DNA +protein |
| Z15331 | M | 4.1 | 6.7 | No | No | DNA +protein |
| Z14289 | M | 5.3 | 11.5 | No | Yes | DNA +protein |
| Z15258 | M | 4.3 | 8.7 | No | Yes | DNA +protein |

^a^Only animals originating from areas endemic for Coccidioidomycosis were tested prior to study enrollment

^b^DNA and protein vaccine regimen comprised of HBV core and surface antigens and anti-CD180

Abbreviations: male (M), not determined (N.D.), simian immunodeficiency virus (SIV), Valley Fever (VF)
